# A Comparative Study of the Cardiovascular Effects of a Lower Dose of Heat-Stable Carbetocin Versus the Standard Dose in the Prevention of Postpartum Hemorrhage During Elective Cesarean Delivery: A Single-Blinded, Randomized, Parallel-Group Trial

**DOI:** 10.7759/cureus.65049

**Published:** 2024-07-21

**Authors:** Rajasri G Yaliwal, Neelamma G Patil, Shailaja Bidri

**Affiliations:** 1 Department of Obstetrics and Gynecology, Bijapur Lingayat District Education (Deemed to be University) Shri B. M. Patil Medical College, Hospital and Research Centre, Vijayapura, IND

**Keywords:** cesarean section, high-sensitivity cardiac troponin i, electrocardiogram, blood pressure, pulse

## Abstract

Introduction: Oxytocin is a uterotonic drug that acts on receptors in the myometrium, causing uterine contractions. However, oxytocin receptors are also present in other organs, including the myocardium. Heat-stable carbetocin, a long-acting analog of oxytocin, is also known to act on these oxytocin receptors. As carbetocin has a long half-life of 40 minutes, its duration of action on the myocardium may be relatively longer than that of oxytocin. Therefore, this study aimed to study the cardiovascular effects of using a lower dose of carbetocin (50 mcg) compared to the standard dose (100 mcg) during elective cesarean delivery.

Materials and methods: A total of 212 full-term pregnant women were randomized into two groups: group I received 50 mcg of intravenous carbetocin, and group II received 100 mcg of intravenous carbetocin. Heart rate, blood pressure (BP), oxygen saturation, electrocardiogram changes, and pre- and postoperative (12 hours after cesarean delivery) high-sensitivity cardiac troponin I levels were compared between the groups.

Results: No statistically significant differences were observed between the groups with respect to heart rate, BP, electrocardiogram changes, or difference in pre- and postoperative high-sensitivity cardiac troponin I levels (p* *> 0.05).

Conclusion: Carbetocin's cardiovascular effects were similar in both groups. None of the participants had adverse cardiovascular effects from the drug, and there were no differences in cardiovascular effects between the groups.

## Introduction

Postpartum hemorrhage (PPH), which is most commonly caused by uterine atony, is the leading cause of maternal mortality globally. Therefore, the World Health Organization has advised the prevention of uterine atony by the administration of uterotonic drugs. Heat-stable carbetocin, an oxytocin analog, is a relatively new drug used for the prevention of PPH. Introduced in 2018, carbetocin has been considered only for the prevention of PPH, and its use in treating PPH has not been advised or licensed [1,2]. In 2021, carbetocin became commercially available in India.

Heat-stable carbetocin is a uterotonic drug whose mechanism of action is similar to that of oxytocin; its longer duration of action resulting from its long half-life of 40 minutes makes it possible to administer it as a single dose during elective cesarean delivery. Oxytocin, however, is recommended as an infusion for a few hours after the elective cesarean delivery. The action of oxytocin on myocardial receptors present in the heart is a known cause of myocardial ischemia. However, as oxytocin has a short half-life, this effect is rarely observed. However, due to the longer half-life of carbetocin, this effect could be hypothetically enhanced, with potentially prolonged cardiovascular effects [3].

Currently, there is no consensus on the recommended dose of uterotonics for either elective or emergency cesarean delivery. Various studies have shown that lower doses of oxytocin and carbetocin are as effective as standard doses in elective cesarean section [4]. Therefore, the above-mentioned potential for adverse cardiovascular effects may be reduced without decreasing the effectiveness of carbetocin if a lower dose is used.

This study aimed to compare the cardiovascular effects resulting from a relatively lower dose of carbetocin versus a standard dose in the prevention of PPH during elective cesarean section. The differences in heart rate (HR), blood pressure (BP), oxygen saturation (SpO_2_), electrocardiograph (ECG) changes, and high-sensitivity cardiac troponin I levels before and after the administration of carbetocin were assessed in both groups.

## Materials and methods

This study was conducted in the Department of Obstetrics and Gynecology, Bijapur Lingayat District Education (Deemed to be University) Shri B. M. Patil Medical College, Hospital and Research Centre, Vijayapura, Karnataka, India. The inclusion criteria for the study were as follows: participants had to be experiencing a full-term pregnancy (i.e., 37-42 weeks of gestation), be over 18 years of age, be undergoing elective cesarean delivery under subarachnoid block (spinal anesthesia), and have provided consent to take part in the study. Patients were excluded from the study if they were in labor, had predisposing medical and/or obstetric conditions for PPH, or had cardiovascular, hepatic, or renal diseases or epilepsy.

We obtained ethical clearance to perform the study from the Institutional Ethics Committee (reference no. BLDE [DU]/IEC/575/2021-22) in accordance with the Declaration of Helsinki. The study was registered with Clinical Trials of India (CTRI/2022/03/041222).

Sample size

**Figure 1:**
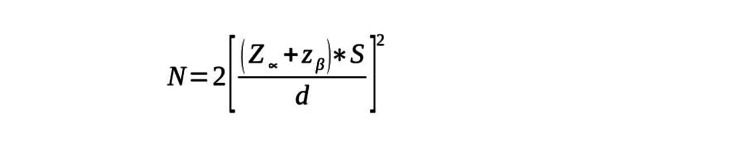
Formula for calculating sample size

The anticipated mean blood loss for carbetocin at doses of 40 and 100 mcg was 806 ± 310 and 697 ± 453 mL, respectively, as per a study conducted by Khan et al. [5]. A minimum sample size of 212 was required to determine any statistically significant differences between the two groups while achieving an explanatory power of 80% and a level of significance of 5% (two-sided) [5], where Z_∝_ is the level of significance (95%), z_β_ is the power of the study (80%), d is the difference between two clinical parameters, and S is the standard deviation (Figure 1).

Statistical analysis

The data obtained were entered into a Microsoft Excel spreadsheet, and statistical analysis was performed using SPSS version 21 (IBM Corp., Chicago, IL). Results are presented as means, counts, percentages, and diagrams. Normally distributed continuous variables between the groups were compared using independent t-tests, whereas the Mann-Whitney U test was used to compare nonnormally distributed variables. Categorical variables between two groups were compared using the chi-square test. A p value of <0.05 was considered statistically significant. All statistical tests were two-tailed.

Study design

The study was a single-blinded, randomized, parallel-group trial. Randomization was done via a computerized randomization chart obtained from www.randomizer.org.

Data collection

A total of 2,553 women who visited the labor ward at Bijapur Lingayat District Education (Deemed to be University) Shri B. M. Patil Medical College, Hospital and Research Centre, were screened for eligibility to participate in the study. Finally, the study included 212 women admitted to the labor ward and fulfilled the inclusion criteria. All participants provided written informed consent before participating in the study.

Preoperative HR and BP were recorded for each patient. ECG and high-sensitivity cardiac troponin I blood tests were performed. Each participant was assigned to a group via a computer-generated randomization chart. The participants were blinded to the dose of carbetocin that they received, whereas the operating obstetrician and anesthesiologist were not blinded to the patient’s group assignment. Cesarean delivery was done under spinal anesthesia with inj. bupivacaine (0.5%) and 60 mcg of inj. buprenorphine.

Women assigned to group I were administered 50 mcg of carbetocin as a bolus dose IV within one minute of the delivery of the neonate. Women assigned to group II were administered 100 mcg of carbetocin IV within one minute after the delivery of the neonate. All doses were administered over the course of one minute.

HR, BP, and SpO_2_ were recorded 1, 5, and 10 minutes after administration of carbetocin and at the end of the procedure. An ECG was performed one hour after the procedure. High-sensitivity cardiac troponin I was tested 12 hours after the procedure.

## Results

**Figure 2:**
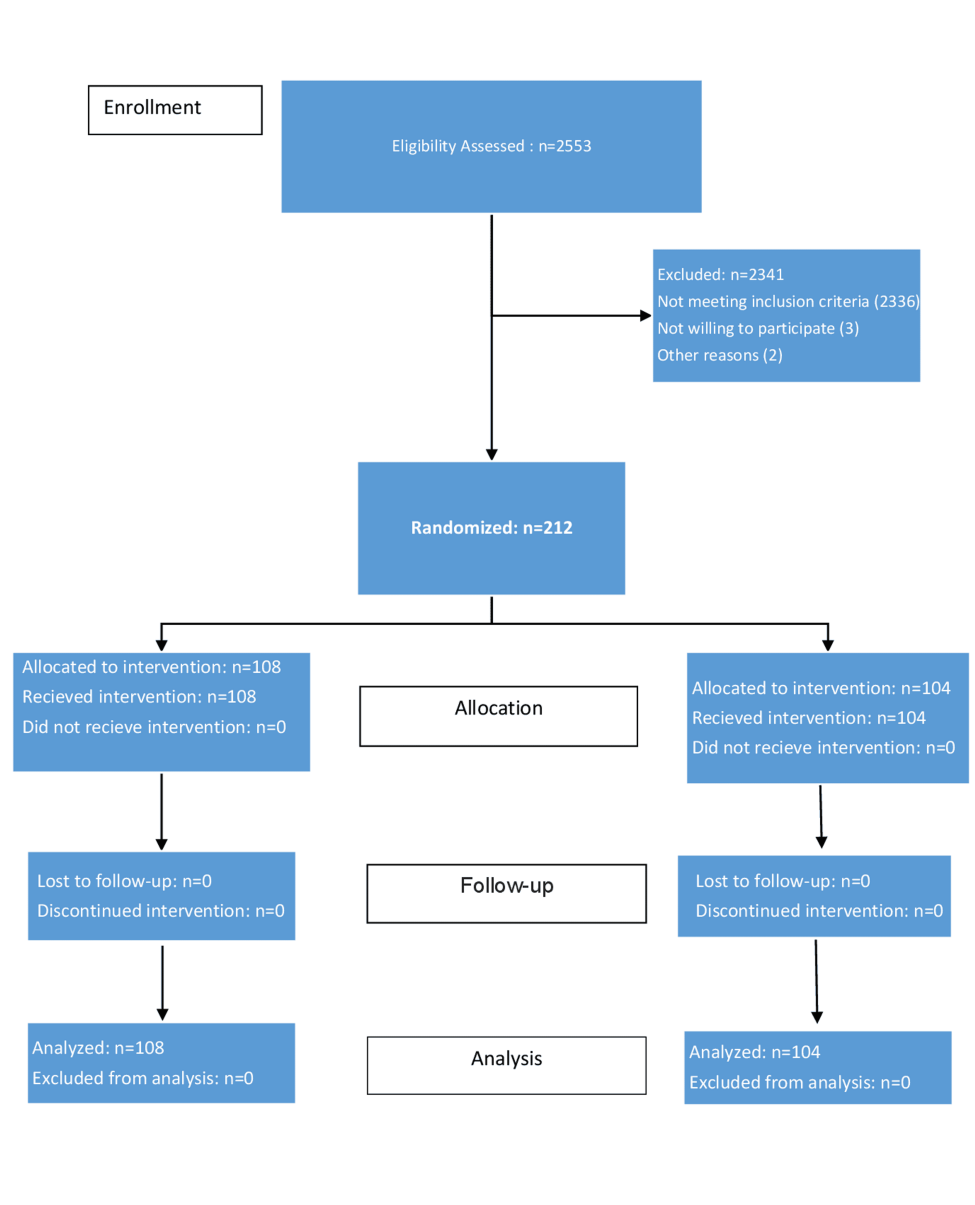
Consort flow diagram

A total of 2,553 women delivered during the study period (July 2022 to January 2023). Out of these, 212 were included in the study and randomized to either group I or II, as shown in Figure 2.

No significant difference was observed in HR between the groups before the intervention or at one and five minutes after the intervention. However, HR showed statistically significant differences between the groups 10 minutes after the intervention despite remaining within a clinically normal range. HR was within a normal range in both groups at the end of the procedure, as shown in Table 1.

**Table 1:** Heart rate data for groups I and II p < 0.05 is considered statistically significant

Heart rate (beats per minute)	Group	Frequency (n)	Mean	Standard deviation	95% confidence interval for mean		Man-Whitney U test	p value
					Lower bound	Upper bound		
Immediately before intervention	I	108	91.49	13.925	88.83	94.15	5,525.000	0.838
	II	104	91.98	15.370	88.99	94.97		
	Total	212	91.73	14.619	89.75	93.71		
At one minute	I	108	92.41	14.574	89.63	95.19	5,384.000	0.603
	II	104	93.21	18.659	89.58	96.84		
	Total	212	92.80	16.668	90.55	95.06		
At five minutes	I	108	93.06	15.760	90.05	96.06	4,979.500	0.154
	II	104	90.17	15.419	87.17	93.17		
	Total	212	91.64	15.624	89.53	93.76		
At 10 minutes	I	108	93.11	15.178	90.22	96.01	4,760.000	0.045
	II	104	89.25	13.914	86.54	91.96		
	Total	212	91.22	14.665	89.23	93.20		
At the end of the procedure	I	108	88.36	13.154	85.85	90.87	5,000.000	0.167
	II	104	85.46	9.697	83.58	87.35		
	Total	212	86.94	11.652	85.36	88.52		

No statistically significant differences in systolic or diastolic BP were observed between the groups before, during, or after the procedure. In addition, there was no significant increase or decrease in either systolic or diastolic BP recordings during the procedure in both groups, as shown in Tables 2, 3.

**Table 2:** Systolic blood pressure data for groups I and II p < 0.05 is considered statistically significant

Systolic blood pressure (mmHg)	Group	Frequency (n)	Mean	Standard deviation	95% confidence interval for mean		Man-Whitney U test	p value
					Lower bound	Upper bound		
Before intervention	I	108	118.62	10.721	116.58	120.67	5,549.000	0.880
	II	104	132.36	14.147	104.84	159.87		
	Total	212	125.36	9.937	111.90	138.81		
At one minute	I	108	118.65	11.506	116.45	120.84	5,329.000	0.519
	II	104	117.63	11.770	115.35	119.92		
	Total	212	118.15	11.620	116.58	119.72		
At five minutes	I	108	119.25	10.211	117.30	121.20	5,410.500	0.644
	II	104	118.67	11.125	116.51	120.84		
	Total	212	118.97	10.647	117.53	120.41		
At 10 minutes	I	108	118.36	9.800	116.49	120.23	5,259.000	0.422
	II	104	117.97	11.040	115.82	120.12		
	Total	212	118.17	10.404	116.76	119.58		
At the end of the procedure	I	108	118.44	10.815	116.37	120.50	5,335.000	0.524
	II	104	117.67	9.900	115.75	119.60		
	Total	212	118.06	10.359	116.66	119.46		

**Table 3:** Diastolic blood pressure data for groups I and II p < 0.05 is considered statistically significant

Diastolic blood pressure (mmHg)	Group	Frequency (n)	Mean	Standard deviation	95% confidence interval for mean		Man-Whitney U test	p value
					Lower bound	Upper bound		
Before intervention	I	108	74.37	11.154	72.24	76.50	5,234.500	0.390
	II	104	75.62	12.859	73.11	78.12		
	Total	212	74.98	12.008	73.36	76.61		
At one minute	I	108	71.59	12.545	69.20	73.99	5,338.000	0.533
	II	104	72.56	12.971	70.04	75.08		
	Total	212	72.07	12.734	70.34	73.79		
At five minutes	I	108	72.08	11.564	69.88	74.29	5,563.500	0.906
	II	104	71.88	11.709	69.60	74.15		
	Total	212	71.98	11.608	70.41	73.55		
At 10 minutes	I	108	70.17	11.063	68.06	72.28	5,348.500	0.548
	II	104	71.69	12.236	69.31	74.07		
	Total	212	70.92	11.650	69.34	72.49		
At the end of the procedure	I	108	74.61	10.083	72.69	76.53	5,457.500	0.720
	II	104	74.43	10.442	72.40	76.46		
	Total	212	74.52	10.237	73.14	75.91		

SpO_2_ remained normal throughout the procedure in both groups, with no statistically significant differences observed after carbetocin administration, as shown in Table 4.

**Table 4:** Oxygen saturation data for groups I and II p < 0.05 is considered statistically significant

SpO_2_ (%)	Group	Frequency (n)	Mean	Standard deviation	95% confidence interval for mean		Man-Whitney U test	p value
					Lower bound	Upper bound		
At one minute	I	108	99.32	4.007	98.56	100.09	5,440.500	0.560
	II	104	99.76	0.512	99.66	99.86		
	Total	212	99.54	2.884	99.15	99.93		
At five minutes	I	108	98.81	8.650	97.16	100.46	5,535.000	0.813
	II	104	99.61	0.781	99.45	99.76		
	Total	212	99.20	6.197	98.36	100.04		
At 10 minutes	I	108	99.62	0.693	99.49	99.75	5,522.500	0.791
	II	104	99.55	0.902	99.37	99.72		
	Total	212	99.58	0.801	99.48	99.69		
At the end of the procedure	I	108	99.64	0.742	99.50	99.78	5,461.000	0.649
	II	104	99.63	0.671	99.49	99.76		
	Total	212	99.63	0.706	99.54	99.73		

High-sensitivity cardiac troponin I tests were done preoperatively and 12 hours postoperatively, as shown in Table 5.

**Table 5:** High-sensitivity cardiac troponin I levels for groups I and II p < 0.05 is considered statistically significant

High-sensitivity cardiac troponin I (ng/dL)	Group	Frequency (n)	Mean	Standard deviation	95% confidence interval for mean		Man-Whitney U test	p value
					Lower bound	Upper bound		
Preoperative cardiac troponin I	I	108	2.652	1.5311	2.360	2.944	5,253.000	0.414
	II	104	4.320	16.2981	1.151	7.490		
	Total	212	3.470	11.4697	1.917	5.023		
Postoperative cardiac troponin I	I	108	3.9796	11.47929	1.7899	6.1694	5,554.500	0.890
	II	104	3.6486	3.98928	2.8727	4.4244		
	Total	212	3.8172	8.63829	2.6477	4.9867		

There were no differences in the ECG traces between the groups recorded preoperatively and one hour postoperatively. No ST segment changes were noted in either group, as shown in Table 6.

**Table 6:** ECG readings for groups I and II p < 0.05 is considered statistically significant ECG: electrocardiograph; QTC: corrected QT interval

ECG parameters	Group	Frequency (n)	Mean	Standard deviation	95% confidence interval for mean		Man-Whitney U test	p value
					Lower bound	Upper bound		
Preoperative parameters								
Heart rate (bpm)	I	108	85.82	13.912	83.17	88.48	5,080.500	0.229
	II	104	86.68	11.502	84.45	88.92		
	Total	212	86.25	12.764	84.52	87.97		
PR interval (ms)	I	108	0.13661	0.043787	0.12826	0.14496	5,024.000	0.172
	II	104	0.14317	0.049957	0.13346	0.15289		
	Total	212	0.13983	0.046919	0.13348	0.14618		
QRS (ms)	I	108	3.85352	16.993846	0.61186	7.09518	5,052.500	0.246
	II	103	3.07095	14.766160	0.18506	5.95684		
	Total	211	3.47151	15.912398	1.31201	5.63100		
QTC (ms)	I	107	0.42931	0.176413	0.39550	0.046312	4,951.500	0.166
	II	104	0.40317	0.198306	0.36461	0.44174		
	Total	211	0.41643	0.187533	0.39098	0.44188		
RV5 (ms)	I	108	11.448566	110.3882658	-9.608540	32.505672	5,543.500	0.871
	II	104	0.822650	0.5477576	0.716125	0.929175		
	Total	212	6.235852	78.7902884	-4.431362	16.903067		
RV5 + SI1 (ms)	I	108	0.59692	0.493991	0.50269	0.69115	4,991.000	0.161
	II	104	0.69490	0.518287	0.59411	0.79570		
	Total	212	0.64499	0.507235	0.57631	0.71366		
Postoperative ECG parameters								
Heart rate (bpm)	I	108	84.01	9.681	82.16	85.86	5,188.000	0.336
	II	104	85.71	11.645	83.45	87.98		
	Total	212	84.84	10.698	83.40	86.29		
PR interval (ms)	I	108	0.14250	0.130818	0.11755	0.16745	4,548.500	0.015
	II	104	0.15721	0.074514	0.14272	0.17170		
	Total	212	0.14972	0.106972	0.13523	0.16420		
QRS (ms)	I	108	4.2582	17.06897	1.0023	7.5142	5,236.000	0.393
	II	104	3.6644	15.48544	0.6529	6.6760		
	Total	212	3.9669	16.27553	1.7634	6.1704		
QTC (ms)	I	1,080	0.45085	0.183481	0.41585	0.48585	5,214.500	0.368
	II	104	0.43838	0.0.208985	0.39774	0.47903		
	Total	212	0.44474	0.196038	0.41819	0.47128		
RV5 (ms)	I	108	0.90822	0.555131	0.80233	1.01412	5,288.500	0.463
	I	104	1.01989	0.855173	0.85358	1.18620		
	Total	212	0.96300	0.0718612	0.86571	1.06030		
RV5 + SV1	I	108	0.78370	0.594200	0.67036	0.89705	5,419.500	0.660
	II	104	0.80300	0.600500	0.68622	0.91978		
	Total	212	0.79317	0.595960	0.71248	0.87386		

## Discussion

This study was conducted to compare the cardiovascular effects of heat-stable carbetocin administered at the standard dose and a relatively lower dose during cesarean delivery. Heat-stable carbetocin has promising applications in places where cold-chain facilities are unavailable, as oxytocin requires a cold chain, and its effectiveness is reduced when the chain is broken. The main drawback of carbetocin is its cost [6].

Our study found no significant differences in HR, BP, SpO_2_, or ECG changes between groups I and II. These results suggest that carbetocin may not act similarly on myocardial receptors as it does on oxytocin receptors. High-sensitivity cardiac troponin I levels remained similar pre- and postoperatively in both groups. Rabow et al. studied cardiovascular changes in women who received 100 mcg of carbetocin versus 5 international units of oxytocin during elective cesarean delivery, concluding that there was no significant difference between groups with respect to SpO_2_, HR, BP, or ECG; however, they did observe higher aggregate vasopressor use in the carbetocin group [7]. Oxytocin, which is known to act on oxytocin receptors present in the heart, also acts on vasopressin receptors due to the similarity in its structure to vasopressin. In contrast, carbetocin does not act on vasopressin receptors. Other uterotonics, such as methergine and carboprost, also act on the cardiovascular system [8].

In a double-blind, randomized, controlled trial, Bahr et al. compared the effects of IV oxytocin and carbetocin administered to 80 women (40 per group) [9]. A significant increase in HR and decrease in BP from baseline to all intervals (i.e., 1, 5, 10, and 15 minutes after administration) were seen in both groups. The oxytocin group exhibited a significantly higher increase in HR, as well as a decrease in BP and SpO_2_, compared to the carbetocin group, suggesting the use of carbetocin for women with hypertensive disorders of pregnancy. Pisani et al. compared the cardiovascular effects of oxytocin and carbetocin during cesarean section, concluding that their effects were similar [10].

The time interval between the peak and end of the T wave measures the dispersion of myocardial repolarization. In a study done by Clunies-Ross et al., although administration of 50 mcg of carbetocin was associated with less transmural dispersion of myocardial repolarization compared to a dose of 100 mcg, this difference did not have any known clinical significance [11]. In our study, we excluded hypertensive patients, as carbetocin, licensed in India, states hypertension as a contraindication for its use. Tabl et al. compared carbetocin doses of 20-100 mcg during elective cesarean delivery, concluding that lower doses were not inferior to the standard dose with respect to blood loss, uterine tone, HR, and BP [12]. Another study comparing bolus administration of carbetocin to infusion concluded that the former could increase HR, making the latter a safer option in women with cardiovascular disease [13].

The ability of carbetocin to prevent PPH seems promising. In a meta-analysis studying the effect of carbetocin on PPH prevention, carbetocin required fewer additional drugs to treat PPH, with less blood loss than syntometrine, making it a promising alternative to oxytocin [14]. The results of our study showed that a lower dose of carbetocin could potentially be a safer option than the standard dose. Carbetocin is becoming the preferred drug for cesarean delivery, especially in women who have conditions that predispose them to PPH [15]. However, additional studies are required to determine the optimal dose and adverse effects of the drug. Lower doses could also bring down the cost of the drug.

Study limitations

In this study, continuous ECG monitoring of all leads with Holt-Oram monitoring was not feasible. Although ECG leads were connected during the cesarean delivery and monitored by a team of anesthesiologists, it was difficult to record the readings for documentation. However, no significant ECG changes were observed in any of the participants. Another limitation is that the sample size is small.

## Conclusions

In this study, a lower dose of carbetocin exhibited similar cardiovascular effects as the standard dose in the prevention of PPH during elective cesarean section. The standard dose of carbetocin did not reveal any untoward cardiovascular effects. Use of a lower dose of carbetocin could lower costs.
